# A Novel Virus for Croup

**DOI:** 10.1371/journal.pmed.0020274

**Published:** 2005-08-23

The World Health Organization estimates that about 20% of all deaths in children younger than five years old are due to acute lower respiratory tract infections (LRTIs), with 90% of these deaths due to pneumonia. But despite LRTIs being among the most frequent diseases in the first years of life, the viral causes of these illnesses are not always clear. Several viruses are known to be involved, e.g., respiratory syncytial virus (RSV), influenza viruses, parainfluenza viruses, and human metapneumovirus, but none of these pathogens is detected in a substantial number of cases.

In a new article published in *PLoS Medicine*, Lia van der Hoek and colleagues investigate the association of acute LRTIs with human coronavirus HCoV-NL63, a virus they recently described. They suggest that HCoV-NL63, a new member of the Coronaviridae family, is one of the most frequently detected viruses in children less than three years old with LRTIs and that this virus is strongly associated with croup.

The team analyzed samples from the PRI.DE study, a prospective population-based study of LRTIs in children younger than three years old in Germany. They assessed by PCR 949 samples of nasopharyngeal secretions from children with LRTIs.

In all, 49 samples (5.2%) were positive for HCoV-NL63 RNA. Viral RNA was more prevalent in samples from outpatients (7.9%) than hospitalized patients (3.2%), and co-infection with either RSV or parainfluenza virus 3 was observed frequently. With an overall occurrence of 5.2%, HCoV-NL63 was the third most frequently detected pathogen in this patient group (RSV was the highest, found in 31.4% of samples). The researchers focused on HCoV-NL63 in cases of respiratory disease where no other viral pathogen could be detected in order to identify clinical symptoms associated with HCoV-NL63 infection. Samples in which only HCoV-NL63 RNA could be detected had a significantly higher viral load than samples containing additional respiratory viruses. A strong association with croup was apparent: 43% of the HCoV-NL63-positive patients with high HCoV-NL63 load and absence of co-infection had croup, compared with 6% in the HCoV-NL63-negative group. Indeed, a significantly higher fraction (17.4%) of samples from croup patients than non-croup patients (4.2%) contained HCoV-NL63 RNA.

This study strengthens the evidence for the role of HCoV-NL63 in croup. Previous studies have shown croup to occur mostly in boys, with peak occurrence in the second year of life and predominantly in the late autumn or early winter season. And HCoV-NL63 infection seems to follow these trends, said the authors.

However, the authors warned that the high percentage of HCoV-NL63-positive samples could be due to a strong viral activity in the study year, and long-term studies are needed to determine whether HCoV-NL63 infections occur in cycles peaking every two to three years, as observed for other respiratory viruses. But they noted that HCoV-NL63 has spread worldwide, with the virus found in Australia, Canada, Japan, Belgium, and the US. Thus, health authorities should add HCoV-NL63 to the list of pathogens that can cause numerous LRTIs in young children.

